# Association between Nine Types of TCM Constitution and Five Chronic Diseases: A Correspondence Analysis Based on a Sample of 2,660 Participants

**DOI:** 10.1155/2017/9439682

**Published:** 2017-06-01

**Authors:** Yanbo Zhu, Huimei Shi, Qi Wang, Yangyang Wang, Xiaohan Yu, Jie Di, Xiaomei Zhang, Yanni Li, Tong Li, Hui Yan

**Affiliations:** ^1^School of Management, Beijing University of Chinese Medicine, Beijing 100029, China; ^2^School of Preclinical Medicine, Beijing University of Chinese Medicine, Beijing 100029, China

## Abstract

**Objective:**

The purpose of this study was to explore the association of nine types of Traditional Chinese Medicine (TCM) constitution with the five chronic diseases: hypertension, hyperlipidemia, diabetes mellitus, heart disease, and obesity.

**Methods:**

Chi-squared test was performed to investigate the distribution characteristics of TCM constitutions in the participants with the five chronic diseases in questionnaire. Correspondence analysis was used to explore the correlation between them.

**Results:**

A total of 2,660 participants (1,400 males; 1,260 females) were included in this study. The mean age was 52.54 ± 13.92. Of them, 600 were of gentleness type accounting for 22.56%. Proportions of gentleness type in the chronic diseases (16.00%~23.70%) were less than that in general population (32.14%). The gentleness type and yin-deficiency type were significantly correlated with hypertension and diabetes mellitus, qi-deficiency type was correlated with heart disease, phlegm-dampness type was associated with obesity, and dampness-heat type was correlated with hyperlipidemia.

**Conclusions:**

The correlations between TCM constitution types and the five chronic diseases were different. This may have a significant implication for TCM practice, and even the people with gentleness type should not be ignored in health management.

## 1. Introduction

Chronic diseases such as cardiovascular disease-heart disease, hypertension, and stroke-cancer, diabetes, and chronic respiratory disease are the main causes of death [1]. The rapid growth of chronic diseases brings heavy burden on healthcare and society. The major risk factors of these diseases come from tobacco smoking, alcohol consumption, unhealthy diet, and lack of physical activity [1–3].

In terms of the theory of Traditional Chinese Medicine (TCM), TCM constitution, depending on the intrinsic characteristics of human body, is innate and influenced by the environment. It integrates the morphological structure and physiological function with psychological state. Although relatively stable, TCM constitution may also develop and change so as to adapt to the changes in the environment throughout a human body's life [4]. The TCM constitutions are classified into nine categories: gentleness type, qi-deficiency type, yang-deficiency type, yin-deficiency type, phlegm-dampness type, dampness-heat type, blood-stasis type, qi-depression type, and special diathesis type. The classification is defined in terms of physical features, common manifestations, psychological characteristics, susceptibility to certain diseases, and adaptability to the environment [5].

Previous studies have shown that unhealthy status [6, 7] or diseases like diabetes mellitus and cardiovascular diseases were correlated with specific constitution types [8–12]. It has been found that more likely to suffer from angina pectoris are the constitutions of dampness-heat type, phlegm-dampness type, and blood-stasis type commonly found in Lingnan area in the southern part of China [12]. It also has been demonstrated that some health conditions and disease status can be improved by the interventions according to TCM constitution [13–16]. These findings suggest that the constitution types found in people with chronic diseases may provide valuable information for disease prevention and treatment [17]. However, the correlation between TCM constitution types and chronic diseases was still unclear.

Number of cases and its proportion as well as binary logistic regression were often used to evaluate the correlation between TCM constitution types and some diseases [8, 10, 11]. However, when analyzing categorical variables like the nine types of TCM constitution and the five chronic diseases, these methods are difficult to reveal the correlation between them. In this study, a multivariate statistical method- correspondence analysis (CA) was applied to investigate the correlation of TCM constitution types in participants with the five chronic diseases.

## 2. Materials and Methods

### 2.1. Subjects

The data of this study was obtained from a cross-sectional survey of 21,218 subjects from December 2005 to January 2007 [18]. By using purposive sampling method, nine provinces and municipalities in China (Jiangsu, Anhui, Gansu, Qinghai, Fujian, Beijing, Jilin, Jiangxi, and Henan) were selected. In each province and municipality, the subjects were collected from communities, health examination centers, or colleges. Systemic sampling method was used to select subjects. All the participants signed an informed consent form.

In our study, the eligible subjects were the participants with self-reported chronic diseases: hypertension, hyperlipidemia, diabetes mellitus, heart disease, and obesity. A number of 2,660 participants (1,400 males and 1,260 females) were included in this study and 18,558 individuals who have none of the five chronic diseases were excluded.

### 2.2. Measurement of TCM Constitution

TCM constitutions were measured and classified with Constitution in Chinese Medicine Questionnaire (CCMQ) [19–21]. CCMQ is a self-rating scale with good reliability and validity. It has 60 items, which contains nine subscales: gentleness, qi-deficiency, yang-deficiency, yin-deficiency, phlegm-dampness, dampness-heat, blood-stasis, qi-depression, and special diathesis. Score of each subscale is standardized from 0 to 100. Gentleness type is a balanced one with higher score indicating a better constitution status, while the other eight types are pathological types with higher score indicating worse constitution status.

Discrimination analysis was applied to determine the constitution type of each subject based on the data from 542 subjects with typical constitutions diagnosed by TCM constitution experts [22].

### 2.3. Correspondence Analysis

It is a statistical method to analyze two-way and multiway category data that are transformed into cross tables [23–26]. In CA, the relationship between the categories of the same variable and the correspondence among different variables can be revealed. The results of CA are demonstrated graphically in a biplot with rows and columns as points, and the interpretation is based upon proximities between points. The procedure of biplot analysis is as follows [27]. First, the proximity of each category of the same variable on horizontal and vertical axes of the biplot should be compared. If they are in close proximity on horizontal or vertical axis, it means there are little differences among them on that axis. Second, the proximity among categories of different variables should be compared. If the categories are in close proximity, they are associated with each other. The closer the proximity, the stronger the association.

### 2.4. Statistical Methods

Chi-squared test and CA were performed to identify the relationship between nine TCM constitution types and the five chronic diseases. Data analysis was conducted with SPSS 17.0 (SPSS Inc., released 2008, SPSS for Windows, Version 17.0, Chicago, IL, USA) and the critical value of statistical significance was set as 0.05.

## 3. Results

### 3.1. Clinical Characteristics of the Participants

Totally 2,660 participants (52.63% males and 47.37% females) were included in this study. The mean age was 52.54 ± 13.92 years, and 1,188 (44.66%) participants were 55 years and above. Of the 2660 participants, 600 were in gentleness type, accounting for 22.56%; the numbers of participants with hypertension, hyperlipidemia, diabetes mellitus, heart disease, and obesity were 1,466 (55.11%), 911 (34.25%), 412 (15.49%), 650 (24.44%), and 351 (13.20%), respectively (Table 1).

### 3.2. Distribution of TCM Constitution Types among the Participants with the Five Chronic Diseases

There were differences among the distributions of TCM constitution types in different chronic diseases (*χ*^2^ = 138.31, *P* < 0.001). The proportions of gentleness type in the chronic diseases varied from 16.00% to 23.70%, which were less than that in general population (32.14%) [22]; the proportions of special diathesis type for each chronic disease were less than the others; and the proportions of qi-deficiency type were higher than the others in chronic diseases except obesity (Table 2).

The top three pathological constitution types in the five chronic diseases were qi-deficiency type (19.00%), phlegm-dampness type (10.90%), and yin-deficiency type (9.10%) in hypertension participants; qi-deficiency type (17.30%), phlegm-dampness type (14.70%), and dampness-heat type (10.20%) in hyperlipidemia participants; qi-deficiency type (19.20%), dampness-heat type (11.90%), and phlegm-dampness type (11.40%) in diabetes mellitus participants; qi-deficiency type (30.80%), yang-deficiency type (11.10%), and blood-stasis type (9.40%) in heart disease participants; phlegm-dampness type (19.90%), qi-deficiency type (15.70%), and dampness-heat type (11.10%) in obesity participants (Table 2).

### 3.3. Correspondence Analysis

**Figure 1:**
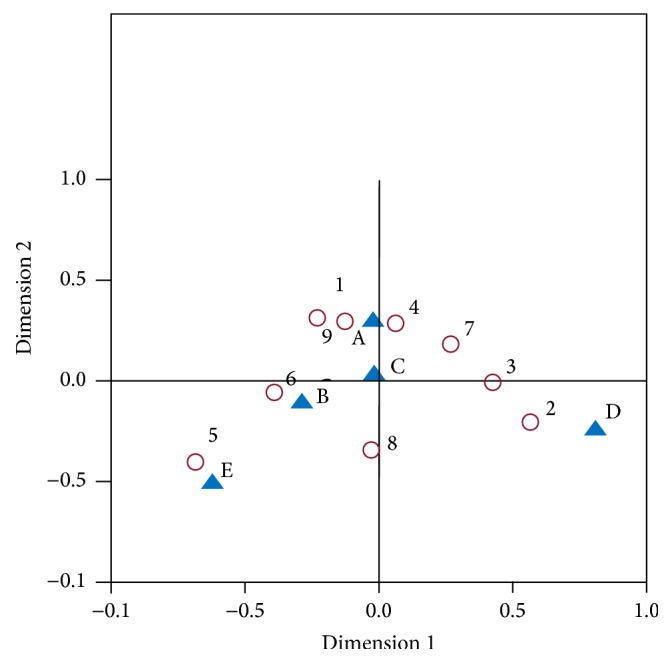
Correspondence analysis biplot of TCM constitution types and five chronic diseases. ○: TCM constitution types, 1: gentleness type, 2: qi-deficiency type, 3: yang-deficiency type, 4: yin-deficiency type, 5: phlegm-dampness type, 6: dampness-heat type, 7: blood-stasis type, 8: qi-depression type, and 9: special diathesis type; ▲: chronic diseases, A: hypertension, B: hyperlipidemia, C: diabetes mellitus, D: heart disease, and E: obesity.

The results of CA are presented in Tables 3 and 4 and Figure 1. From Table 3, the singular value indicates the correlation between the row and column profiles. The eigenvalues of dimension 1 and dimension 2 were 0.778 and 0.139, respectively, and 91.70% of the variance was represented well in the first two dimensions (Table 3).

The factor loading matrix of constitution types and chronic diseases on dimension 1 and dimension 2 was showed in Table 4, and it was also graphically showed in a biplot (Figure 1). Since dimension 1 (77.80%) revealed much more information than dimension 2 (13.90%), we can only see the results on dimension 1 (horizontal axis of Figure 1). On dimension 1 of Figure 1, the nine TCM constitution types fell into two categories: (1) 1 (gentleness type), 5 (phlegm-dampness type), 6 (dampness-heat type), 8 (qi-depression type), and 9 (special diathesis type); (2) 2 (qi-deficiency type), 3 (yang-deficiency type), 4 (yin-deficiency type), and 7 (blood-stasis type). The five chronic diseases were also divided into two categories: (1) A (hypertension), B (hyperlipidemia), C (diabetes mellitus), and E (obesity); (2) D (heart disease). According to the spatial distribution of variables, gentleness type and yin-deficiency type were correlated with hypertension and diabetes mellitus; qi-deficiency type was associated with heart disease; phlegm-dampness type was correlated with obesity; and dampness-heat type was correlated with hyperlipidemia.

## 4. Discussion

Different chronic diseases have different distribution of TCM constitutions according to the constituent ratios. But it is difficult to clearly explain the distribution of TCM constitution types in chronic diseases. CA is a multivariate statistical analysis method used to explore the relationship between several variables. It is based on the analysis of the contingency table through the row and column profiles to present a unique graphical display showing the relationship among variables [28]. Therefore, on the basis of constituent ratios, CA was used to reveal the relationship between chronic diseases and TCM constitutions.

Results showed that hypertension and diabetes mellitus were related to yin-deficiency type. Hypertension is recognized as dizziness, headache, liver yang, and liver wind in TCM. The main characteristics of yin-deficiency type are the consumption of body fluids, essence and blood, and the deficiency of internal heat [5]. It is recognized in* Internal Canon of Huangdi* that deficiency of kidney essence and hyperactivity of liver yang may lead to dizziness. Meanwhile, some study [29] has found that, in hypertension participants with yin-deficiency and yang hyperactivity, the renin, angiotensin II, and serum lipid peroxidation significantly increased, and plasma nitric oxide and atrial natriuretic factor significantly decreased, which may relate to the development of hypertension.

The diabetes mellitus is recognized as consumptive thirst in TCM. It is documented in* Jingyue Quanshu • Zazhengmo • Xuezheng* that all blood syndromes result from insufficiency of fluids leading to fire flaring up regardless of zang-organs. It is more likely to suffer from consumptive thirst with excessive fire flaring up due to insufficiency of yin for a long time.

Interestingly, we found that gentleness type was also related to hypertension. The characteristics of gentleness type are moderate posture, shiny and glossy complexion, and vigorous and good function of zang-fu organs. In this research, the mean age of the participants was 52.54, and about half of them were 55 years old and above. One possible explanation was that hypertensive participants with gentleness type may have a better survival rate, which may justify the association of gentleness type with hypertension. Another possible explanation was that people with gentleness type believe that they are in good physical condition and pay no attention to their unhealthy behavior and habits like drinking and smoking [30], which increases hypertension prevalence in the gentleness type participants.

In addition, we found that obesity was related to phlegm-dampness type, and hyperlipidemia was related to dampness-heat type. Phlegm-dampness type resulted from accumulation of phlegm due to internal retention of dampness. Previous study has indicated that phlegm-dampness type and qi-deficiency type are the main constitution risk factors of overweight or obesity [31]. Dampness-heat type is a constitution type of internal retention of dampness-heat, which is an important pathogenesis of hyperlipidemia according to both TCM traditional literatures and modern studies [32]. Studies have also found that dampness-heat type is associated with metabolic syndrome [33, 34].

Our findings also show that qi-deficiency was related to heart disease. In TCM, heart disease is recognized as heart pain, which is due to qi blockage in the chest. Throughout the process of qi blockage in the chest, heart qi is deficient [35]. Qi acts as the commander of blood while blood is the mother of qi, which means that qi manages the generation and circulation of blood and blood is the carrier of qi. The deficiency of heart qi can result in heart disease due to slow or impeded flow of blood.

There are some limitations in this study. First, this study was based solely on cross-sectional data. Longitudinal studies should be conducted to confirm the findings. Second, the chronic diseases were self-reported and only five chronic diseases were involved in the study. More chronic diseases are recommended to be evaluated in further studies. Third, TCM constitution is also correlated with other factors like eating habits, geological locations, and climates [36]. It would be better to recruit more participants in different areas and to further analyze the correlations between TCM constitution types and chronic diseases in the future.

## 5. Conclusions

In summary, the associations of TCM constitution types differ in the participants with the five different chronic diseases. Yin-deficiency type and gentleness type were associated with hypertension and diabetes mellitus; phlegm-dampness type was correlated with obesity; dampness-heat type was associated with hyperlipidemia; and qi-deficiency type was correlated with heart disease. Therefore, different interventions based on the TCM constitution should be conducted for different chronic diseases preventions and interventions. At the same time, people with gentleness type should not be ignored in health management.

## Figures and Tables

**Table 1:** Clinical characteristics of 2,660 patients.

Characteristic	Classification	*n*	Percentage (%)
Gender	Male	1,400	52.63
	Female	1,260	47.37
Age (years)	15–24	85	3.20
	25–34	190	7.14
	35–44	495	18.61
	45–54	702	26.39
	55–64	573	21.54
	≥65	615	23.12
Education	Primary school	324	12.18
	Middle school	1,267	47.63
	College degree and above	1,069	40.19
Constitution types	Gentleness type	600	22.56
	Qi-deficiency type	508	19.10
	Yang-deficiency type	230	8.65
	Yin-deficiency type	225	8.46
	Phlegm-dampness type	295	11.09
	Dampness-heat type	248	9.32
	Blood-stasis type	216	8.12
	Qi-depression type	194	7.29
	Special diathesis type	144	5.41
Chronic diseases	Hypertension	1,466	55.11
	Hyperlipidemia	911	34.25
	Diabetes mellitus	412	15.49
	Heart disease	650	24.44
	Obesity	351	13.20

**Table 2:** Distribution of nine types of TCM constitution in five chronic diseases.

Chronic diseases	*n*	Gentleness type	Qi-deficiency type	Yang-deficiency type	Yin-deficiency type	Phlegm-dampness type	Dampness-heat type	Blood-stasis type	Qi-depression type	Special diathesis type
A	1466	348 (23.70)	279 (19.00)	117 (8.00)	134 (9.10)	160 (10.90)	127 (8.70)	121 (8.30)	94 (6.40)	86 (5.90)
B	911	206 (22.60)	158 (17.30)	65 (7.10)	61 (6.70)	134 (14.70)	93 (10.20)	73 (8.00)	70 (7.70)	51 (5.60)
C	412	85 (20.60)	79 (19.20)	43 (10.40)	34 (8.30)	47 (11.40)	49 (11.90)	30 (7.30)	24 (5.80)	21 (5.10)
D	650	104 (16.00)	200 (30.80)	72 (11.10)	52 (8.00)	43 (6.60)	40 (6.20)	61 (9.40)	49 (7.50)	29 (4.50)
E	351	74 (21.10)	55 (15.70)	21 (6.00)	28 (8.00)	70 (19.90)	39 (11.10)	18 (5.10)	29 (8.30)	17 (4.80)

A: hypertension, B: hyperlipidemia, C: diabetes mellitus, D: heart disease, and E: obesity; *χ*^2^ = 138.31, *P* < 0.001.

**Table 3:** Statistics summary of correspondence analysis.

	Singular value	Inertia	Proportion of inertia	
			Accounted for	Cumulative
Dimension				
1	0.168	0.028	0.778	0.778
2	0.071	0.005	0.139	0.917
3	0.046	0.002	0.057	0.974
4	0.031	0.001	0.026	1.000
Total		0.036	1.000	1.000

**Table 4:** Factor loading matrix of correspondence analysis.

Variable	Dimension 1	Dimension 2
Constitution types		
Gentleness type	−0.230	0.310
Qi-deficiency type	0.566	−0.204
Yang-deficiency type	0.423	−0.011
Yin-deficiency type	0.059	0.286
Phlegm-dampness type	−0.685	−0.404
Dampness-heat type	−0.391	−0.060
Blood-stasis type	0.264	0.181
Qi-depression type	−0.032	−0.343
Special diathesis type	−0.127	0.295
Chronic diseases		
Hypertension	−0.024	0.293
Hyperlipidemia	−0.289	−0.111
Diabetes mellitus	−0.021	0.031
Heart disease	0.809	−0.249
Obesity	−0.623	−0.513
